# Clinical assessment of complementary treatment with *Qishen Yiqi* dripping pills on ischemic heart failure: study protocol for a randomized, double-blind, multicenter, placebo-controlled trial (CACT-IHF)

**DOI:** 10.1186/1745-6215-14-138

**Published:** 2013-05-14

**Authors:** Ya Zhu Hou, Shuai Wang, Zhi Qiang Zhao, Xian Liang Wang, Bin Li, Shan Bin Soh, Jing Yuan Mao

**Affiliations:** 1 Tianjin University of Traditional Chinese Medicine, 312 Anshan Western Road, Tianjin, Nankai District, China; 2 Cardiovascular Department of The First Teaching Hospital of Tianjin University of Traditional Chinese Medicine, 314 Anshan Western Road, Tianjin, Nankai District, China; 3 National Clinical Research Center of Traditional Chinese Medicine on Coronary Artery Disease, Tianjin, China

**Keywords:** 6-minute walking test, Ischemic heart failure, *Qishen Yiqi* dripping pills, Traditional Chinese medicine

## Abstract

**Background:**

Heart failure (HF) is associated with decreased quality of life, high re-admission rate and poor prognosis. In particular, ischemic heart failure (IHF) has a worse prognosis than nonischemic HF. The use of traditional Chinese medicine (TCM) alongside Western medicine to treat HF has developed into an integrative treatment model in China. There have been small clinical trials and experimental studies to demonstrate the efficacy of TCM for treating HF; however, there is still a lack of high-quality trials. *Qishen Yiqi* dripping pills (QSYQ), a TCM drug, have been commonly used alongside standardized Western medicine to treat IHF. This paper describes the protocol for the clinical assessment of QSYQ in IHF patients.

**Method:**

A randomized, double-blind, multicenter, placebo-controlled trial will assess the efficacy and safety of QSYQ in the treatment of IHF. The trial is to enroll 640 IHF patients from 32 hospitals in China. Besides their standardized Western medicine, patients will be randomized to receive treatment of either QSYQ or placebo for 6 months and follow-up monitoring for at least a further 6 months. The primary outcome is increased exercise capacity of patients, which will be measured using the 6-minute walking test (6MWT). The secondary outcomes include composite endpoints: all-cause mortality, frequency of hospitalization or emergency due to cardiovascular events, brain natriuretic peptide levels, left ventricular ejection fraction, and cardiothoracic ratio will be documented, as well as scores on the New York Heart Association classification and Minnesota quality of life index, and information obtained from the four TCM diagnostic methods. Blood lipid tests will also be administered.

**Discussion:**

The integrative treatment model of TCM alongside Western medicine has developed into a treatment model in China. The rigorous design of the trial will assure an objective and scientific assessment of the efficacy and safety of QSYQ in the treatment of IHF.

**Trial registration:**

Clinical trials.gov number: NCT01555320

## Background

Heart failure (HF), as the endstage of various cardiac diseases, is associated with decreased quality of life, high re-admission rate, and poor prognosis. Some investigations showed that ischemic heart disease (IHD) was responsible for 70% of HF patients, while 40% of IHD patients died from HF [1-3]. Even with standardized treatment, the prognosis of patients with ischemic heart failure (IHF) is worse than in patients with nonischemic HF, especially for those with severe symptoms (New York Heart Association (NYHA) class IV) [4]. Their 1-year and 3-year mortality rates are 18% and 43%, respectively [4]. Nowadays, Western medicine treatment for HF is standardized around the world [5,6]; however, the integrative treatment of Western and traditional Chinese medicine (TCM) for HF is commonly used and developed as a treatment model in China [6].

*Qishen Yiqi* dripping pills (QSYQ, produced by Tasly Pharmaceutical Co. Ltd.), a patent drug made of active ingredients from *Huangqi*, *Danshen*, *Sanqi*, and *Jiangxiang,* has the effect of nourishing *Qi*, promoting blood circulation and relieving pain. The pills have been commonly used in TCM for the integrative treatment of patients with IHF who are also diagnosed with *Qi* deficiency and blood stasis syndrome [7,8]. Experimental researches have shown in animal models that QSYQ could inhibit platelet aggregation, prevent enlargement of end-diastolic diameter, improve left ventricular function, and slow down ventricular remodeling [9-14]. Some small-scale clinical studies on the drug demonstrated efficacies in improving left ventricular function, increasing exercise capacity and decreasing re-admission rate [15-19].

We would like to test the hypothesis that patients with IHF will benefit from TCM treatment QSYQ and evaluate its safety through a high-quality clinical trial.

### Objective

The objective of the trial is to assess the safety and clinical effects of QSYQ in improving the exercise capacity of IHF patients.

## Design

The CACT-IHF study is designed as a multicenter, randomized, double-blind, placebo-controlled trial, and will be conducted in 32 level-A hospitals in China. A total of 640 stable outpatients with IHF, who fulfill the inclusion and exclusion criteria, will be randomized into either a treatment group or a control group.

### Sample size

The sample size calculation was based on results from studies by Hutcheon *et al*. [20] and Mao *et al. *[21]. After 6 months of intervention, we assume that the distance walked in a 6-minute walking test (6MWT ) in the control group and treatment group will be up to 260 m and 288 m, respectively (that is, patients in the treatment group will walk an additional 28 m, *δ* = 115 m). Patients will be randomized in the ratio of 1:1 into two groups and each group requires at least 266 patients in order to produce positive results (one-tailed test, significant level *α* = 0.05, power (1 - *β*) = 0.80). Considering the loss of patients due to follow-up and a maximum dropout rate of less than or equal to 20%, the sample size was increased to 320. To calculate sample size, we used the equation:

$$n=\frac{2{\left(Z_{1-\alpha }+Z_{1-\beta }\right)}^{2}\delta^{2}}{{\left(\mu_{T}-\mu_{C}\right)}^{2}}$$

### Inclusion criteria

• Age 40 to 79 years.

• Patients with IHF:

◦ Left ventricular ejection fraction ≤45%, as measured by echocardiography in modified Simpson method.

◦ History of myocardial infarction with or without percutaneous coronary intervention or coronary artery bypass grafting.

◦ Coronary angiography or coronary computed tomography angiography shows ≥50% stenosis in at least one main coronary artery with or without revascularization, which the researcher thinks is closely related to HF.

◦ With or without dyspnea, fatigue and fluid retention (edema), and so on.

• History of HF or present with HF symptoms for at least 3 months.

• NYHA Class II to IV.

• Submitted informed consent.

### Exclusion criteria

• Acute HF or acute exacerbation of chronic HF.

• Patients with one of the following diseases:

◦ Acute coronary syndrome within 30 days.

◦ Revascularization therapy within 6 months.

◦ Uncontrolled hypertension with systolic pressure ≥180 mmHg or diastolic pressure ≥110 mmHg.

◦ Second-degree type 2 or worse sinoatrial or atrioventricular block without implantation of pacemaker or uncontrolled malignant cardiac arrhythmia.

◦ Dilated cardiomyopathy.

◦ Hypertrophic obstructive cardiomyopathy.

◦ Myocarditis.

◦ Pulmonary artery embolism.

◦ Severe valvular heart disease

◦ Pulmonary heart disease.

◦ Stroke within 6 months.

• Cardiac resynchronization therapy.

• Applied diuretics, cardiotonic agents, or vasodilators intravenously within 7 days.

• Severe endocrine diseases, such as hyperthyroidism.

• History of malignant tumor.

• Hemoglobin ≤ 9 g/dl.

• Alanine aminotransferase more than twice the upper limit of normal.

• Serum creatinine >265 μmol/l.

• Mental disorder.

• Pregnant, planning for pregnancy or breastfeeding.

• Suspicious or definite allergy to intervention drugs.

• Participated in other trials within 2 months.

• Unable to walk autonomously, owing to physical disabilities.

### Randomization procedure

Included patients will be randomly assigned to a treatment or control group in the ratio of 1:1. Stratification will be performed on NYHA classification, enrolling hospitals, and revascularization methods. Eligible patients will be randomized by IWRS/IVRS software (ClinicalSoft Co. Ltd.) into the intervention arms. The identification number and randomization code generated are unique to each patient and correspond to the coded boxes of either QSYQ or a placebo matched in appearance, color, and shape to the drug. The flavor of the drug and placebo are also similar. Blinding will be maintained amongst the investigators and patients.

### Interventions and study plan

Written informed consent will be obtained from all eligible patients before enrolment. Subsequently, all enrolled patients are screened for 10 ± 3 days (V0). During this period, their Western medication will be standardized according to their clinical conditions with reference to the 2010 HFSA comprehensive heart failure practice guideline [5] and 2007 CSC guidelines for the treatment and diagnosis of chronic heart failure [6]. The standardized Western medications include diuretics, angiotensin-converting enzyme inhibitor or angiotensin-receptor blocker, β-blockers, aldosterone receptor antagonist, digoxin, and vasodilating agents. To achieve a heart-healthy lifestyle, patients will also be advised to restrict salt and water intake, monitor weight, and maintain regular physical activities.

After screening, stable patients will be randomized to either a treatment or control group at the baseline visit (V1). Besides their standardized Western medicine, patients assigned to the treatment or control group will take QSYQ or placebo, respectively. Patients have to take one packet (0.52 g) of the study medication thrice daily, with the first dose taken as soon as practically possible. Independent drug administrators will be responsible for the delivery and dispensation of the study medications.

**Figure 1:**
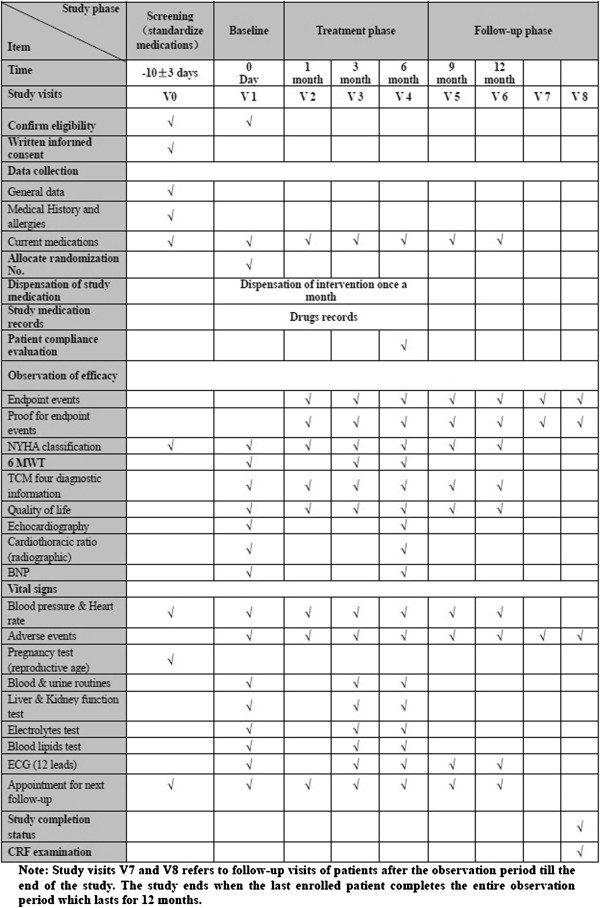
Study visits.

This trial has two phases: treatment and follow-up. In the 6-months course of treatment, study medications will be given to the patients, who are to attend study visits at 1, 3, and 6 months (V2, V3, and V4). After the treatment phase, patients will enter a follow-up phase lasting for at least another 6 months. During this follow-up period, patients are to attend study visits at 9 and 12 months (V5 and V6), and two additional study visits during extended follow-up (V7, V8). The extended follow-up ends when the last patient enrolled into the trial completes 12 months of treatment and follow-up. All the visits and contents are described in Figure 1.

During the entire trial period, any other kinds of TCM drugs are forbidden except for QSYQ or the placebo. The study medication should be taken routinely for 6 months unless disallowed because of certain clinical conditions. In the case of comorbidities (such as hypertension or diabetes) and complications, appropriate Western medicine can be given with reference to the relevant guidelines. All drugs used should be recorded up to date by the investigators. Patients should not hesitate to seek emergency care if necessary.

### Outcomes

#### Primary outcome

The 6MWT will be used to assess the exercise capacity of the patients in this trial [22]. This test will be used for each patient at study visits V1, V3, and V4, and conducted in hospitals on a 30-meter track. The patients will be instructed to walk as far as possible for 6 minutes, at a self-determined speed and pausing to rest as needed. Before each test, the patient’s blood pressure, heart rate, and oxygen saturation will be measured. Subsequently, effort perception on the Borg scale will also be assessed. At the end of each test, the same parameters will be measured again. The result of the 6MWT is the total distance covered (in meters) in 6 minutes.

#### Secondary outcomes

Composite endpoints, including all-cause mortality, frequency of hospitalization or emergency due to cardiovascular events (exacerbated HF, acute coronary syndrome, malignant ventricular arrhythmias, cardiac shock, revascularization, stroke, pulmonary embolism, peripheral vascular events, and so on) will be documented at study visits from V2 to V8. Additional secondary outcome measures, such as brain natriuretic peptide levels, echocardiography (including left ventricular ejection fraction quantified by the Simpson method) and cardiothoracic ratio by radiography will be included for the patients at study visits V1 and V4. The NYHA classification and Minnesota quality of life scores, as well as information from the four TCM diagnostic methods, will be documented at study visits from V1 to V6.

#### Other measurements

Blood lipid tests (blood cholesterol, triglycerides, high-density lipoprotein, and low-density lipoprotein) will be carried out at study visits V1, V3, and V4.

#### Safety

Safety will be assessed by vital signs, laboratory tests, ECG, and adverse events. Vital signs, including heart rate and blood pressure, will be monitored at enrolment and study visits V1 to V6. Laboratory tests, such as routine blood and urine tests, hepatic and renal function, and blood electrolyte levels will be examined at study visits V1, V3, and V4. Electrocardiograms will be recorded at study visits V1, V3, V4, V5, and V6. Adverse events will be documented at every study visit and severe adverse events will be reported to the principal investigator within 24 hours. In the case of a medical necessity, the patients will be unblinded according to the unblinding procedures on receiving permission from the principal investigator. A negative pregnancy test is also mandatory before enrolment of women of reproductive age.

**Figure 2:**
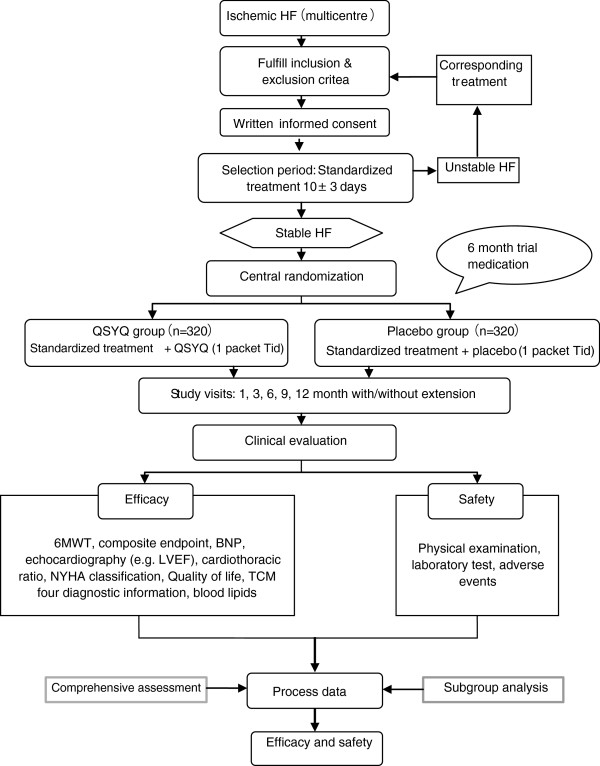
Schedule of evaluations.

A schedule of all evaluations is outlined in Figure 2.

### Data collection

Patients will be followed up by a study investigator until the end of trial, and all general data and outcome evaluations will be recorded in the case report form at each study visit. Investigators will also update an electronic database using Oracle Clinical Remote Data Capture. This will provide real-time monitoring of the database, to ensure the high quality of data. At the end of the trial, the investigators, data managers, and statisticians will perform blind review and confirm the database before processing data.

### Ethical aspects

This trial protocol was approved by the Ethics Committee of First Teaching Hospital of Tianjin University of TCM on 2 December 2011 (TYLL2011 [K] 005). The protocol and its informed consent form have been judged ethically and scientifically satisfactory for the trial aims. Written informed consent must be obtained from all participants or their legally authorized representatives before enrollment. The trial has been registered with both the Chinese Clinical Trial Registry of International Clinical Trials Registry Platform of World Health Organization and ClinicalTrial.gov of the U.S. National Institutes of Health.

### Statistical analysis

Statistical analysis for the trial will be performed by the Centre for Statistical Science, Peking University. Statistics software SAS 9.1 will be used for the data analysis. Intention-to-treat analysis, per-protocol analysis, and safety analysis will be conducted. Calculations for the outcomes will be based on a two-tailed test, except for the primary outcome, which will be a one-tailed test (level of significance *α* = 0.05). Besides the statistical comparison between baseline variables, the change in 6MWT result between baseline and the assessment 3 and 6 months during the treatment phase will be performed using analysis of covariance adjusted for baseline and treatment to improve the efficiency and power of the tests. Survival analysis will be conducted using Kaplan-Meier and Cox regression models. Statistical analysis on the missing data of the outcome measures will be adjusted using an estimating equation or statistical model in the final analysis. Sensitivity analyses will also be performed to assess the robustness of the data.

## Discussion

The use of TCM alongside Western medicine in the treatment of HF is a common practice in China and has developed into an integrative treatment model. The efficacy of the model to improve the exercise capacity, quality of life, and symptoms of these patients has been demonstrated in some small-scale clinical studies [23-26]. However, there is a lack of high-quality evidence to support the recommendation of integrative treatment for HF.

QSYQ, as a TCM drug often used in IHF treatment, has been studied in many small-scale clinical trials and intensive experimental researches [9-19]. However, there is still a demand for a high-quality and larger-scale clinical research to prove its efficacy in the treatment of IHF. As TCM is composed of multi-active components and possesses multitarget action feature, it can provide a holistic regulation of whole body and improve overall condition of patients. To assess the results of the treatment effectively, an appropriate outcome measure is required. QSYQ has showed its advantage in increasing exercise capacity of IHF patients, therefore the 6MWT, which has been proven to be a good measurement of exercise capacity [27,28], could be a proper primary outcome test for the trial.

The study protocol was designed with reference to international clinical trial principles. Strict quality control measures were implemented and data were monitored regularly. Quality control supervisors will ensure that investigators adhere to the study protocol in each investigation center. The trial has also applied IWRS and Oracle Clinical Remote Data Capture to enhance data management and reduce bias in the research. The rigorous design of the trial will ensure an objective and scientific assessment of the efficacy and safety of QSYQ in the treatment of IHF.

## Trial status

The CACT-IHF study has been recruiting patients since March 2012. Study completion date is estimated at August 2014. Clinical trials.gov number: NCT 01555320.

## Abbreviations

6MWT: 6-minute walking test; HF: Heart failure; IHD: Ischemic heart disease; IHF: Ischemic heart failure; NYHA: New York Heart Association; QSYQ: *Qishen Yiqi* dripping pills; TCM: Traditional Chinese medicine.

## Competing interests

The authors declare that they have no competing interests.

## Authors’ contributions

YZH, ZQZ, XLW, BL and SBS took part in the design of the study and did the literature review, SW was responsible for clinical practice, JYM made substantial contributions to the conception and design of the study and took charge of drafting the study protocol essentially, is the corresponding author for the article. All authors read and approved the final manuscript.
